# Persistent and Transient Airborne MRSA Colonization of Piglets in a Newly Established Animal Model

**DOI:** 10.3389/fmicb.2018.01542

**Published:** 2018-07-13

**Authors:** Kerstin Rosen, Uwe Roesler, Roswitha Merle, Anika Friese

**Affiliations:** ^1^Institute for Animal Hygiene and Environmental Health, Freie Universität Berlin, Berlin, Germany; ^2^Institute for Veterinary Epidemiology and Biostatistics, Freie Universität Berlin, Berlin, Germany

**Keywords:** livestock, ST398, pig, aerosol chamber, swine, antibiotic resistance

## Abstract

Livestock-associated methicillin-resistant *Staphylococcus aureus* (LA-MRSA) was first found in 2005 and is up to date widespread in animal husbandry reservoir – focusing on pig farming. The regular detectability of MRSA in the air of pigsties as well as in exhaust air of pig farms (mean count: 10^2^ cfu/m^3^) poses the question whether an airborne spread and, therefore, a MRSA colonization of animals *via* the airborne route exists. To answer this question, we exposed three groups of nine MRSA-negative tested piglets each to a defined airborne MRSA concentration (10^2^, 10^4^, and 10^6^ cfu/m^3^) in our aerosol chamber for 24 h. In the following observation period of 21 days, the MRSA status of the piglets was monitored by taking different swab samples (nasal, pharyngeal, skin, conjunctival, and rectal swab). At the end of the experiment, we euthanized the piglets and investigated different tissues and organs for the spread of MRSA. The data of our study imply the presence of an airborne MRSA colonization route: the animals exposed to 10^6^ cfu/m^3^ MRSA in the air were persistent colonized. The piglets exposed to an airborne MRSA concentration of 10^4^ cfu/m^3^ were transient, and the piglets exposed to an airborne MRSA concentration of 10^2^ cfu/m^3^ were not colonized. Consequently, a colonization *via* the airborne route was proven.

## Introduction

For more than a decade, it is known that the occurrence of methicillin-resistant *Staphylococcus aureus* (MRSA) is no longer restricted to the well-known hospital-acquired MRSA (HA-MRSA) and community-acquired MRSA (CA-MRSA). Livestock – and of outstanding importance – pig farming is a reservoir where MRSA was first described in 2005 (Voss et al., 2005). To distinguish it from the already known MRSA, these variants were referred as livestock-associated MRSA (LA-MRSA). Most of the LA-MRSA isolates are assigned to the clonal complex (CC) 398 and the sequence type (ST) 398 with the predominant *spa* type t011. LA-MRSA is not limited to farm animals anymore and could also be found in companion animals like cats and dogs as well as horses (Vincze et al., 2014). In the last years, there was an increase of MRSA isolates associated to livestock in hospitals of rural areas (Becker et al., 2017).

The main transmission route of MRSA is direct contact to animals as well as living or non-living vectors. In several studies, these resistant bacteria were also found in the air of pig barns as well as in exhaust air of pig farms (Pletinckx et al., 2011; Friese et al., 2012; Ferguson et al., 2016). Furthermore, Ferguson et al. (2016) reported deposited MRSA in soil up to a distance of 215 m of the pig farm surrounding. The spread of MRSA into neighboring farms by the airborne way is a more likely scenario. However, the role of an airborne transmission between animal farms is still unclear. To investigate the possibility of a colonization of piglets through MRSA contaminated air, three groups of piglets were experimentally exposed to different MRSA concentrations as defined aerosol in an aerosol chamber. This study aimed to determine the concentration of airborne MRSA needed for a transient or a persistent MRSA carriage of piglets. Until now, MRSA transmission models are quite artificial with regard to MRSA transmission (nasal drop-in, oral inoculums) or taking a long time for obtaining colonized piglets (colonization of piglets at birth by vaginal MRSA-positive sows). Therefore, in the present study, a new model was also established for colonization of MRSA in piglets through airborne route. For the first time, MRSA colonization was conducted with conventional raised non-antibiotic-treated piglets habiting a common bacterial flora such as methicillin-sensitive *Staphylococcus aureus* (MSSA) in a model that imitates the field conditions of a transmission of MRSA *via* the airborne route as far as possible.

## Materials and Methods

The animal study was permitted by the State Office of Health and Social Affairs Berlin, Germany (Landesamt für Gesundheit und Soziales; number 0403/12).

### Study Design

**FIGURE 1:**
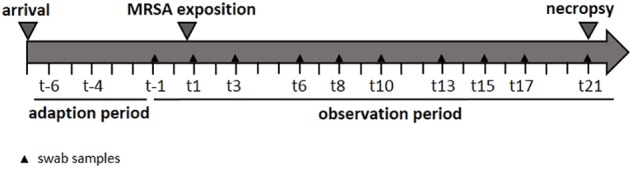
Study design.

In order to determine the specific dose necessary for a successful airborne MRSA colonization of piglets, three groups were exposed once to a defined MRSA concentration in the air for 24 h using an aerosol chamber. The first group was exposed to 10^2^ colony forming units (cfu)/m^3^ (low dose group: LD), the second group to 10^4^ cfu/m^3^ (mid dose group: MD), and a third one to 10^6^ cfu/m^3^ (high dose group: HD). A control group (CT) was treated equally and housed for 24 h in the chamber without any MRSA exposure. The MRSA concentration used for the LD group is equal to the mean MRSA concentration found in the barn air of pigsties (Friese et al., 2012). The MD group was exposed to an airborne MRSA concentration of 10^4^ cfu/m^3^ MRSA – according to Friese et al. (2012) the highest MRSA concentration that was detected in the air of pig farms. Before the exposure in the aerosol chamber, all groups had a 7-day period of acclimatization. Thereafter, the piglets were sampled three times a week. These swabs were analyzed in order to determine the MRSA colonization (see **Figure 1**). Additionally, the environment of the kept pigs was investigated. After an observation period of 21 days, the piglets were euthanized, and different tissues and organs were analyzed qualitatively and quantitatively for the presence of MRSA.

To verify the airborne MRSA dosage that resulted in a transient MRSA colonization of the piglets, the MD group (10^4^ cfu/m^3^) was repeated. The animals were treated equally to the other groups.

### Aerosol Chamber

**FIGURE 2:**
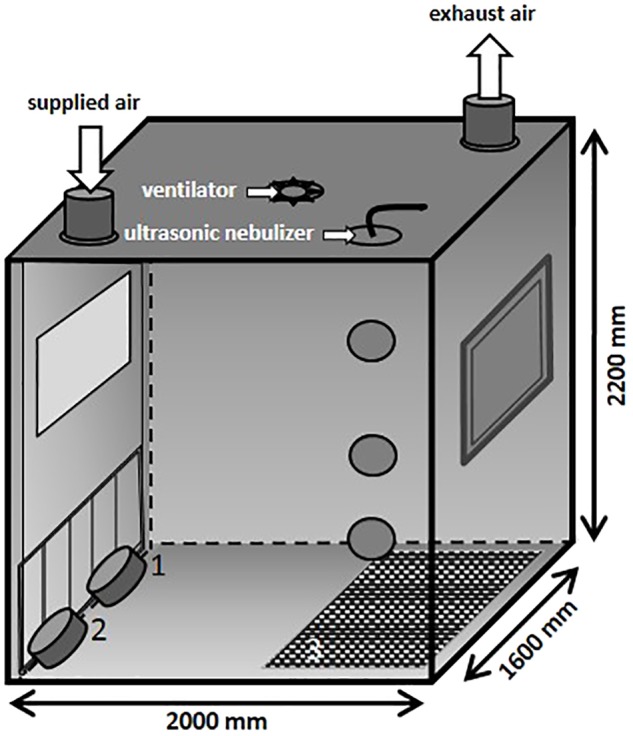
Schematic illustration of the aerosol chamber.

In the present study, an aerosol chamber (**Figure 2**) made of stainless steel was used to generate bio aerosols under defined climatic parameters (relative humidity of 70%, temperature of 26°C and an air flow of 100 m^3^/h). The chamber has a base area of 3.2 m^2^ and a volume of 7 m^3^. The MRSA suspension was aerosolized by using a perfusion pump in combination with an ultrasonic nebulizer (Broadband Ultrasonic Generator, Sono-Tek Corporation, Milton, MA, United States) integrated into the ceiling. The perfusion rate of the pump was adapted to the different desired MRSA concentrations in the air. In the ceiling of the aerosol chamber is one port for exhaust air and one entry port for applying fresh air. The aerosol was dispersed by an axial ventilator situated in the center of the ceiling. Air samples were taken using impingement at different levels: 1.6 m (high position: HP), 0.8 m (middle position: MP), and 0.3 m (low position: LP; exposure level of the piglets) above the ground.

The particle size distribution in the air was measured by an aerosol spectrometer monitor (Grimm, model 1.109, GRIMM Aerosol Technik Ainring GmbH & Co., KG, Germany). During the animals’ exposure, the chamber contained rubber mats (3) that covered half of the ground as well as two troughs for water (1) and feed (2) positioned on the ground and fixed to the chamber door (see **Figure 2**), respectively. The piglets were moving around freely during the whole exposure time.

Air sampling was done using an AGI-30 impinger (Zinsser Analytic, Frankfurt am Main, Germany), filled with 30 mL of phosphate-buffered-saline (PBS; Oxoid, Wesel, Germany). It was connected to the aerosol chamber *via* probes made of steel. The air was sampled for 30 min using an air flow of 12.5 L/min. The flow was verified by using a rotameter. In previous studies, we validated the different MRSA concentrations found in the air of the chamber to reach the required bacterial loads for the animal trials. Therefore, we aerosolized MRSA suspensions under the defined parameters of 26°C, 70% relative humidity, and an airflow of 100 m^3^/h. The MRSA suspension was adapted until the targeted concentration in the air was achieved. The final MRSA suspensions were tested at least three times to confirm the reproducibility. To test the aerosol distribution within the chamber, the MRSA concentration was measured *via* impingement in the three different heights: HP, MP, and LP.

### Bacterial Strain and Preparation of MRSA Suspension

The MRSA strain of the ST 398 was originally provided by the “Federal Institute for Risk Assessment” (BfR) and was isolated from a healthy pig. This MRSA ST 398 strain (strain ID: BfR 08S00974, ITU 1179) was used by Szabó et al. (2012), thereby establishing a nasal colonization model for LA-MRSA.

The MRSA suspension was prepared as follows: first, 100 μl of the specific MRSA culture, that was aerobically incubated overnight in Mueller Hinton broth (Oxoid, Wesel, Germany) with the addition of 6.5% NaCl (MHB+) in a shaking incubator (Multitron, Infors HT, Germany), was plated onto blood base agar (Blood Agar Base No. 2, Oxoid, Wesel, Germany) and was incubated for 8 h at 37°C to achieve the exponential growth phase. Afterward, all colonies of one plate were suspended in 3 mL PBS and homogenized using glass beads and vortexing for 3 min. The MRSA suspension was adjusted to 0.5 McFarland standard by adding PBS to receive a concentration of approx. 1 × 10^8^ cfu/mL. This bacterial concentration was confirmed by measuring the optical density at 600 nm (OD600; OD600 needed to be a value between 0.073 and 0.11) and counting the bacteria using the Neubauer chamber (C-Chip Neubauer improved, Carl Roth GmbH + Co., KG, Karlsruhe, Germany). The suspension was diluted with PBS to gain the predefined specific concentration for the subsequent aerosolization as defined in the preliminary studies.

The MRSA suspension needed for the experiment was divided into portions of 50 mL and loaded in syringes. For the animal exposure, which lasted 24 h, eight syringes with MRSA suspension was used and they were stored on ice until usage. For the LD and MD groups, a new syringe with suspension was applied every 4 h, for the HD group every 75 min due to a higher necessary perfusion rate.

### Animals and Animal Housing

This study included 36 weaned, gender mixed piglets at an age of approximately 21 days. Three groups with nine piglets each were used to determine the MRSA dose needed for a possible airborne colonization, another group of nine animals served as a control group. The animals were housed in the experimental animal facility of the Centre for Infection Medicine of the Department for Veterinary Medicine of Freie Universität Berlin. A strict hygiene regime was performed concerning the entry facility and the used experimental pig barn. The barn was cleaned every day. Manure was removed and the floor was cleaned with water. The staff being in contact with the piglets as well as the pig barn and the aerosol chamber were confirmed MRSA-negative prior to the experiments. Protective clothing was used including snoods and respiratory masks and was changed each time. During the observation period, behavior and health condition were observed daily, and rectal temperature at every sampling time point and the weight of the piglets were monitored weekly.

### Samples

#### Aerosol Chamber

The aerosol chamber was disinfected prior to the animals’ exposure. After that, the walls and floor of the aerosol chamber were sampled using PBS-moistened cotton gauze swabs to confirm the negative MRSA status.

The MRSA concentration in the air was measured during the entire aerosol exposure of the piglets at three time points: 1, 9, and 17 h after starting the aerosolization. Therefore, two air samples (HP and MP) were taken simultaneously using impingement as described above. The lowest sampling location (LP) in the height of the animals was not used due to the risk of injuries to the animals and to avoid an influence of results.

Furthermore, a potential presence of MRSA on the walls of the aerosol chamber after the 24 h exposure and after removing the animals was analyzed. For this, an area of 900 cm^2^ on two chamber walls at a height of 1.5 m was sampled using PBS-moistened cotton gauze swabs.

#### Animal Samples

Nasal, pharyngeal, conjunctival, skin, and rectal swabs were collected the day before and after the MRSA exposure and then three times a week for 21 days.

For the skin and the rectal samplings, cotton swabs with a diameter of 5 mm (nerbe plus GmbH, Winsen, Germany) were used. The skin swab was moistened with PBS. All other samples were taken with sterile dry 3 mm cotton swabs (nerbe plus GmbH, Winsen, Germany). The nasal colonization was examined by scrubbing on the nasal mucosa of both nostrils consecutively in a depth of about 1 cm. The pharyngeal swab was taken by opening the piglets’ mouth and scrubbing the pharynx. For investigating the fecal shedding of MRSA, a dry cotton rectal swab was taken. To determine the skin’s colonization with MRSA, the region behind the ears was swabbed three times on every site. The conjunctival status was investigated applying a single dry cotton swab on both eyes.

At the end of the experiment, the piglets were necropsied to investigate the occurrence of MRSA in the internal organs. The following organs were examined under sterile conditions: ileocaecal, mandibular and lung lymph nodes, palatine tonsils, tracheal bifurcation, lung, and spleen.

#### Environmental Samples

Environmental MRSA contamination of the experimental pig barn was evaluated using moistened swab samples (diameter 5 mm) of the ground, the walls, the feeding trough, and the water trough as well as the toys. The ground and the wall of the pen were sampled by scrubbing two different locations of approximately 20 cm^2^. The feed trough and water trough as well as the toy were tested on approximately 20 cm^2^ at only one position. All samples were handled individually and processed in laboratory within 2 h after sampling.

### Laboratory Analyses

#### Air Samples

After the sampling, the remaining PBS in the impinger was quantified for the calculation of the total MRSA concentration.

The air samples were processed subsequent to sampling. One hundred microliters of an appropriate dilution was streaked out threefold onto chromatic MRSA screen agar (CHROMagar MRSA, MAST Diagnostica GmbH) and incubated aerobically at 37°C. MRSA was identified phenotypically after 24 h of culturing. The MRSA concentration in the air could be calculated by counting the colonies.

#### Swab Samples

For quantification, the swab samples were extracted in 1.5 mL PBS and vortexed gently, and 600 μl were stored at 4°C (retained samples). The remaining sample fluid as well as the swab was mixed with 9 mL MHB+ (Oxoid, Wesel, Germany). After an incubation of 24 h at 37°C, 1 mL of the MHB+ was transferred into 9 mL of Trypton Soy broth (Oxoid, Wesel, Germany) containing 75 mg/L Aztreonam (Molekula GmbH, Munich, Germany) and 3.5 mg/L Cefoxitin (Altmann Analytik GmbH & Co., KG, Munich, Germany; TSB+) and incubated again at 37°C overnight. Every sample fluid was streaked out onto the chromatic MRSA screen agar using a 10 μl inoculation loop. Five MRSA positive subjected colonies per sample were picked and transferred onto Columbia agar with sheep blood plus (Oxoid, Wesel, Germany), incubated at 37°C overnight and confirmed with MALDI TOF mass spectrometry. In case of positive results after the sample enrichment, 100 μl of the retained samples were plated onto chromatic MRSA screen agar in a threefold approach and quantified as described.

The cotton gauze swabs from sampling the aerosol chamber were vortexed and enriched with 180 mL MHB+ at 37°C in the shaking incubator after retained samples were taken. The following day, 20 mL were transferred into 180 mL TSB+ and incubated again for 24 h. The samples were streaked out onto chromatic MRSA screen Agar. MRSA identification was carried out as described before.

#### Internal Organs

In the laboratory, the samples were processed immediately after the necropsy using the same procedure as previously published by Szabó et al. (2012).

The outer layer of the organs or tissues was decontaminated by flaming with 96% ethanol (except for ileocaecal lymph node and tracheal bifurcation), cut into pieces (excluding tracheal bifurcation), weighed to 10 g, and added with 90 mL MHB+. In case of lower mass, the whole sample was used and diluted 1:10 with MHB+. The specimen was homogenized for 2 min at 200 rpm using a stomacher (stomacher 400 circulator; Seward Limited, West Sussex, United Kingdom). A sample fluid of 1 mL was stored at 4°C and with the remaining sample a two-step enrichment with MHB+ and TSB+ was conducted as described before. Qualitative MRSA-positive samples were quantified and colonies, suspected to be MRSA, were verified as described before.

#### *Spa* Typing of MRSA Isolates

For each group, the *spa* typing was conducted for one isolate of every impinger sample and one MRSA isolate origination from the last positive nasal swab of each animal, respectively. In addition, one isolate of every MRSA-positive tonsil of the HD was *spa* typed. All isolates were confirmed as *spa* type t011 using the PCR according to Kahl et al. (2005). LGC Genomics GmbH performed the sequencing. The sequences were analyzed using BioNumerics version 6.6.

### Statistical Analysis

The software SPSS, version 24 (SPSS, Inc., Chicago, IL, United States) was used to perform the statistical analysis. We used a generalized regression model to estimate the effect of the MRSA concentration in the air on the prevalence of MRSA-positive individuals in population (logistic regression models) or on the number of log cfu per individual sample (linear regression models). Animal and type of swab sample were considered as random factors, while day of sampling was considered as repeated measurements in all models.

The influence of the type of swab sample, the group, and the sampling day as well as their interactions were investigated in one set of models. The influence of group and the type of sample including necropsy only at day 21 were determined in a second set of models. Third, the influence of environmental samples was analyzed in a set of models including type of sampling, group and investigation day as fixed factors.

*p*-Values <0.05 were regarded statistically significant. Model diagnosis included normality tests of residuals and visual investigation of homoscedasticity. Results displayed refer to the multivariable models described above.

## Results

### MRSA Aerosol

**Table 1** shows the MRSA concentration in the air measured *via* impingement. The first rows of this table show the results of the evaluation tests concerning the validation of the three targeted MRSA concentrations for the subsequent animal exposures. The results of the air samplings during the animal exposure of all three groups are included. It shows that the targeted MRSA concentrations in the air were reproducible and that all three groups were exposed to the defined MRSA concentration in the air during the different experiments. The results of impingement, especially the values close to minimum and maximum, indicate a very good reproducibility of the aerosol generation. Furthermore, we achieved a very good distribution of airborne MRSA within the aerosol chamber since there was no difference in MRSA concentration between the different located impingers from the chamber’s top to the ground.

**Table 1:** MRSA concentration in the air in cfu/m^3^ for preliminary tests and the animal exposure for the low dose group (LD), median dose group (MD), and high dose group (HD).

		LD (3 × 10^2^ cfu/m^3^)	MD (3 × 10^4^ cfu/m^3^)	HD (3 × 10^6^ cfu/m^3^)
		
		MRSA in air (cfu/m^3^)	MRSA in air (cfu/m^3^)	MRSA in air (cfu/m^3^)
Validation	Mean	6.4 × 10^2^	3.0 × 10^4^	5.0 × 10^6^
Tests (*n* = 3)	Minimum	2.3 × 10^2^	1.6 × 10^4^	2.8 × 10^6^
	Maximum	1.3 × 10^3^	6.3 × 10^4^	7.5 × 10^6^
Animal	Mean	4.2 × 10^2^	3.6 × 10^4^	5.2 × 10^6^
Exposure (*n* = 3)	Minimum	1.3 × 10^2^	1.6 × 10^4^	3.9 × 10^6^
	Maximum	7.1 × 10^2^	6.3 × 10^4^	6.9 × 10^6^

*The data shown here for the preliminary tests based on three repeated measurements using three impingers in three positions (HP, MP, and LP) for each of the three target dosages. Data on the animal exposure are based on three measurements using two impingers (HP and MP) during the 24 h animal exposure.*

The particle size counted by the Grimm aerosol spectrometer was between 3.1 μm (minimum) and 3.7 μm (maximum) for all groups.

The MRSA-negative status of the aerosol chamber was confirmed before starting every animal exposure by sampling the wall using cotton gauze swabs. After the exposure, MRSA was qualitatively detectable on the chamber wall for the LD and MD groups and quantifiable for the HD group with a concentration of 2 and 0.7 cfu/cm^2^, respectively.

### MRSA in the Animals’ Environment

Methicillin-resistant *Staphylococcus aureus* in the pig barn can act as a source for MRSA re-colonization of the animals. Five different swab samples were taken to determine the MRSA status of different surfaces. The concentration of airborne MRSA during the exposure (*p* < 0.001) as well as the sampling day (*p* = 0.035) had a significant influence on the percentage of MRSA-positive environmental swabs. For the LD group, all environmental samples taken from the barn were negative during the whole observation period after the MRSA aerosol exposure. However, 27% (12/45) of the environmental swabs originating from the MD and 98% (44/45) from the HD group taken after the exposure were MRSA positive during the whole observation period. The type of swab sample did not significantly influence the likelihood of an environmental swab being MRSA positive (*p* = 0.282). In the MD group, most MRSA-positive samples were found the first day after the exposure (t1; *n* = 3/5) with 10 cfu/swab and at the end of the observation period (t21; *n* = 4/5). Thus, positive samples were the ground, the feeding and the water trough and, additionally at day 21, the wall. For the other sampling points, one environmental swab was found to be positive only (mostly the ground) with exception of days 15 and 17 where all samples were negative. A quantification was possible at the beginning of the observation period (t1 and t3) only. Except for the toy at day 18, all environmental samples were MRSA positive within the HD group. There, more than half of the samples were quantifiable. The highest number of MRSA/swab was found in ground samples of the pig barn after the exposure (10^2^ cfu/swab) and decreased to 0.1 cfu/swab at the end of the observation period (t21) – similar to the decrease of the MRSA concentration of all other environmental swabs over time. The MRSA concentration found in the ground samples differs significant from the other environmental swabs (from *p* < 0.0001 to *p* = 0.011).

### Clinical Symptoms

No clinical signs were observed in any group during the whole observation period. The body weight development of the exposed animals was comparable to the animals of the control group.

### Animal Colonization

Piglets of the HD group exposed to 10^6^ cfu/m^3^ airborne MRSA were persistently colonized over the whole observation time. By contrast, piglets of the MD group exposed to 10^4^ cfu/m^3^ airborne MRSA were transiently colonized and piglets of the LD group exposed to 10^2^ cfu/m^3^ airborne MRSA were not colonized. The control group remained MRSA negative for the whole observation period. In general, the sampling day (*p* < 0.001) had a significant influence on the MRSA status of a piglet. Also, the MRSA dosage in the air significantly influenced the MRSA status of the piglets: the probability of the LD group’s animals being MRSA positive at respective sampling points of the observation period was significant lower (*p* < 0.001) compared to the MD group. By contrast, animals of the HD group were at significant higher (*p* < 0.001) risk being MRSA positive during the course of time. Interestingly, the type of sample had no significant effect on the likelihood having a MRSA-positive status within the whole observation period (*p* = 0.414). On the other hand, there was a significant influence on the MRSA status of the pigs (*p* = 0.011) when considering the type of swab sample in the course of time. For example, the likelihood of the skin swab being MRSA positive increased at the end of the observation period compared to the pharyngeal swab (*p* = 0.009).

### Nasel Swabs

**FIGURE 3:**
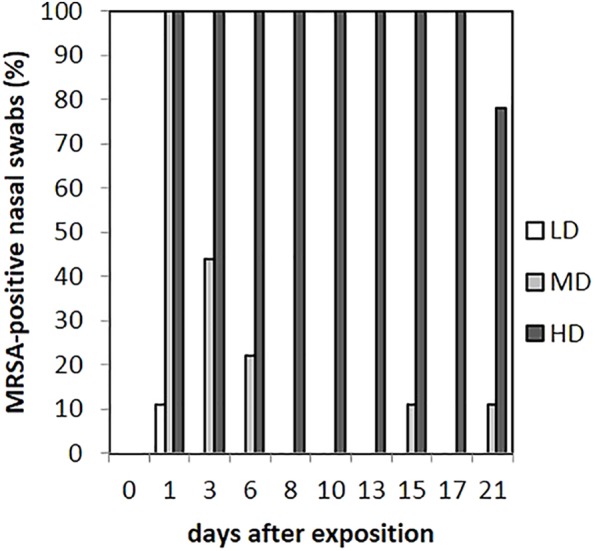
Percentages of MRSA-positive nasal swabs from piglets of the low (LD), mid (MD), and high (HD) dose group over the observation period.

**FIGURE 4:**
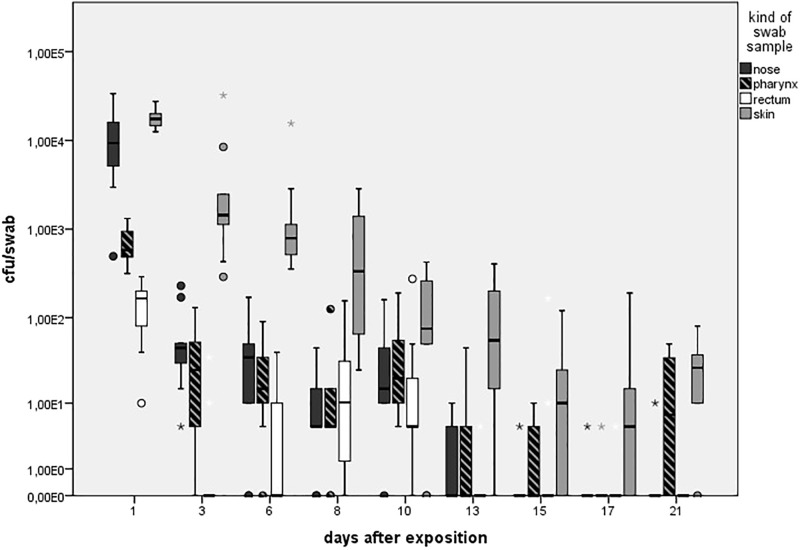
Mean count of quantifiable MRSA-positive swabs (cfu/swab sample) colored in different shades for the high dose group (HD) over the observation period of 21 days. Boxes show the lower quartile, median, and the upper quartile. The ends of the whiskers show the lowest datum within 1.5 interquartile range of the lower quartile and the highest datum within 1.5 interquartile range of the lower quartile and the highest datum within 1.5 interquartile range of the upper quartile. Dots represents outliners. Asterisk represents extreme values. Number of quantifiable swabs for nose/skin/pharyngeal/rectal swabs at day 1 (9/9/9/9), day 3 (9/9/8/3), day 6 (7/9/8/3), day 8 (8/9/7/6), day 10 (8/7/9/7), day 13 (4/8/4/1), day 15 (0/6/3/0), day 17 (1/5/8/1), and day 21 (1/7/5/2).

As presented in **Figure 3**, in the LD group, one animal (*n* = 1/9) showed one MRSA-positive nasal swab directly after the exposure (day 1) only. Then, all nasal swabs were MRSA negative during the whole observation period. For the MD group, all nasal swabs (*n* = 9/9) were MRSA positive directly after the exposure and decreased continuously until day 6 (*n* = 2/9; **Figure 3**). At days 15 and 21, one nasal swab (*n* = 1/9) was MRSA positive, respectively, and isolated from piglet no. 61 (t21) and no. 62 (t15). These animals had MRSA-positive nasal swabs before at days 1 and 3. Animal no. 59 was the only one being MRSA positive for all first three sampling points after exposure. During the entire observation period, 17 out of 81 nasal swabs of the MD group were qualitative MRSA positive, whereas 6 swabs were quantifiable with about 10 cfu/swab sample at day 1. For the HD group, all nasal swabs (*n* = 9/9) were MRSA positive at all sampling times except the last one (**Figure 3**). For that group, **Figure 4** shows the MRSA concentration in the nasal swabs over the observation period. At day 1, all nasal swabs were quantifiable with a mean count of 10^4^ cfu/swab. During the time, the MRSA concentration per swab sample as well as the number of quantifiable samples decreased close to the detectable concentration limit of 5 cfu/swab. At the end of the observation period, quantification was possible sporadically only.

There was a significant difference in the occurrence of MRSA-positive nasal and skin swabs (*p* = 0.011) including the samples from all groups.

Statistical analysis showed that the MRSA concentration of positive nasal swabs over time was twofold higher compared to the pharyngeal swab {*p* < 0.0001; odds ratios (OR) = 2, 141 [95% confidence interval (CI)]}.

### Skin Swabs

**FIGURE 5:**
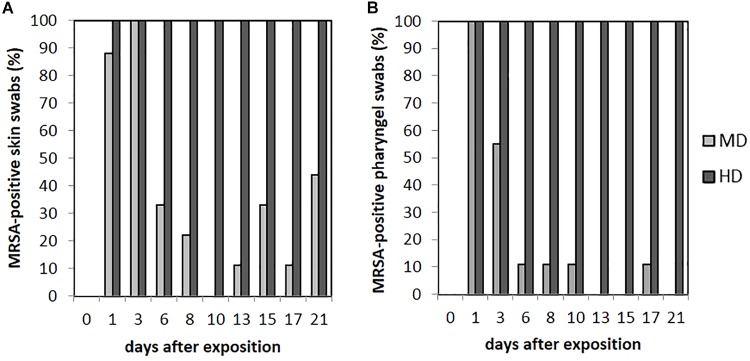
Percentages of MRSA-positive skin **(A)** and pharyngeal **(B)** swabs from piglets of the mid (MD) and high dose (HD) group over the observation period.

As seen in **Figure 5A**, no animal of the LD group had MRSA-positive skin swabs during the whole observation period. 89% (*n* = 8/9) of the skin was tested MRSA positive directly after the exposure for animals of the MD group with an increase to 100% (9/9) at day 3. Then, the MRSA status of the skin varied over the time. Positive skin swabs were detectable until the end of the observation period. In animal no. 61, MRSA could be monitored on the skin for the first four time points as well as day 21. Piglet no. 58 showed positive skin swabs at day 1 as well as day 3. After a period of MRSA-negative skin samples, this piglet became MRSA positive again at the end of the observation period. Quantification of the MD groups’ skin swabs was possible for all qualitative MRSA-positive swabs of day 1 and day 3 with a mean MRSA concentration of 10^2^ cfu/swab. For the HD group, all skin swabs were tested MRSA positive during the whole observation period (see **Figure 5A**). **Figure 4** shows *inter alia* the MRSA concentration of quantifiable skin swabs of the HD group. Quantification was possible for the majority of samples for all sampling points. The mean count decreased from 10^4^ cfu/swab (day 1) to 3.2 × 10 cfu/swab (day 21). Furthermore, comparable to the nasal swabs, the skin swabs’ MRSA concentration was significant higher compared to the MRSA concentration of the pharyngeal swab (*p* < 0.0001; OR = 5.33; 95% CI).

### Pharyngeal Swabs

Within the LD group, all pharyngeal swabs were MRSA negative during the whole observation period. As presented in **Figure 5B** for the MD group, all pharyngeal swabs (*n* = 9/9) were MRSA positive directly after the exposure (day 1) and decreased to 11% (*n* = 1/9) at day 6. Then, MRSA was detected sporadically only. The last positive sample on day 17 originated from piglet no. 63, which was also positive for this kind of swab sample at days 1 and 3. In animal no. 59, MRSA was found in four pharyngeal swabs at days 1, 3, 8, and 10 after the exposure.

All animals of the HD group had MRSA-positive pharyngeal swabs for all sampling points (see **Figure 5B**). Quantification was possible for 67% (*n* = 54/81) of these samples, whereas the mean count was about 10^2^ cfu/swab (*n* = 9/9) directly after the exposure (day 1) and decreased to approximately 10 cfu/swab for the last sampling points (see **Figure 4**).

The presence of MRSA-positive pharyngeal swabs was only significantly lower compared to the skin swabs (*p* = 0.015). The MRSA concentration of the pharyngeal swabs was significantly lower compared to the nasal as well as the skin swabs’ [*p* < 0.0001; OR = 2.141 (nasal swab) and 5.224 (skin swab), 95% CI] MRSA concentration during the time. The MRSA concentration of the pharyngeal swabs was significantly higher (*p* = 0.001; OR = 0.657; 95% CI) compared to the MRSA concentration of the conjunctival swabs.

### Conjunctival Swabs

**FIGURE 6:**
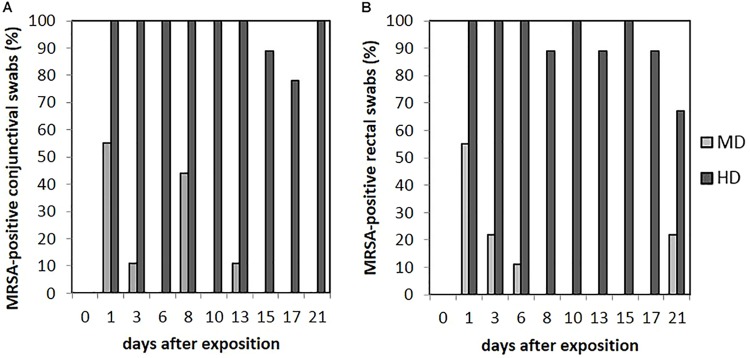
Percentages of MRSA-positive conjunctival **(A)** and rectal **(B)** swabs from piglets of the mid (MD) and high dose (HD) group over the observation period.

Animals of the LD group showed MRSA-negative conjunctival swabs. As shown in **Figure 6A**, MRSA-positive conjunctival swabs of the MD group could be proven at day 1 for 55% (*n* = 5/9) and day 8 for 44% (*n* = 4/9) of the animals. In addition, MRSA-positive conjunctival swabs were seen sporadically. A quantification of these kinds of swab samples was not possible. For the HD group, most of the conjunctival swabs were MRSA positive during the completely observation period (see **Figure 6A**). Here, a quantification was possible until day 9 and, additionally for one sample at day 21. The MRSA load per swab sample was between 5 and 10^2^ cfu/swab. The probability of the conjunctival swabs being MRSA positive was significant lower compared to the skin swabs (*p* = 0.006).

### Fecal Excretion

We did not observe any MRSA-positive rectal swabs within the LD group. As illustrated in **Figure 6B**, the MD group showed MRSA-positive swabs of the rectum the first three sampling points. The detection rate of positive swabs decreased from 55% (*n* = 5/9) at the beginning of the observation period to 11% (*n* = 1/9) at day 6. Apart from day 21 where two piglets had MRSA-positive rectal swabs, the remaining samples were MRSA negative. One out of the two positive samples at day 21 derived from piglet no. 61 that was also positive at day 1; the other positive rectal swab originated from piglet no. 56 that had shown no MRSA-positive swab of this region before. In the HD group, the percentage of MRSA-positive rectal swabs ranged from 89% (*n* = 8/9) to 100% (*n* = 9/9; see **Figure 6B**). All MRSA-negative swabs are attributable to two animals. According to **Figure 4**, the mean MRSA concentration of the rectal swabs was 10^2^ cfu/swab directly after the exposure and decreased over the time. The number of quantifiable samples decreased from 100% directly after exposure (day 1) to sporadic quantification for the end of the observation period. A significant difference between the occurrence of MRSA-positive rectal and skin swabs was found (*p* = 0.006). Considering the other types of swab samples, there was no significant difference (from *p* = 0.073 to *p* = 0.494). In addition, the MRSA load of the rectal swabs was significant lower compared to the nasal, skin, and pharyngeal swabs (from *p* < 0.0001 to *p* = 0.030).

### Internal Organs

In contrast to the HD group, the investigated internal organs of the LD and MD group did not show any MRSA colonization. In animals exposed to the highest MRSA concentration in the air, MRSA was detected in tonsils only. There the bacterial count was between 8.7 × 10^1^ and 2.8 × 10^4^ cfu/tonsil. The MRSA concentration in tonsils was significantly higher compared to all types of animal swab samples taken at day 21 [*p* < 0.0001; from OR = 0.140 (conjunctival swab) to OR = 0.237 (rectal swab); 95% CI].

### Spa Typing

The selected isolates were confirmed as *spa* type t011.

### Second Evaluation of the Mid Dose Group

The MD group was repeated to confirm the transient piglets’ MRSA colonization when exposed to the airborne MRSA concentration of 10^4^ cfu/m^3^. For the second MD group, the mean concentration of MRSA in the air during the animals’ exposure was 4.32 × 10^4^ cfu/m^3^. The number of MRSA-positive swab samples at the respective time points was – for each kind of swab sample – comparable to the MD group previously performed (data not shown). In the same manner as for the first MD group, all investigated internal organs were MRSA negative. The statistical analysis revealed no significant difference (*p* = 0.776) in the probability of the animals being MRSA positive at respective sampling points of the observation period between the two MD groups.

## Discussion

Our study aimed to identify the required dose for a successful (experimental) MRSA colonization of piglets *via* the airborne transmission route. The MRSA concentration in the air required for a long-term colonization was 10^6^ cfu/m^3^ for an exposure time of 24 h. Furthermore, an exposure to 10^4^ cfu/m^3^ resulted in a transient colonization of the animals. The exposure to the lowest used MRSA dose of 10^2^ cfu/m^3^ did not lead to a colonization. Statistical analysis underlines these differences in the MRSA detection when comparing the three animal groups in the course of time. Therefore, it is very likely that an airborne colonization route exists depending on the MRSA concentration in the air.

In the past, several models for an experimental MRSA colonization were conducted. However, all studies used more or less artificial methods for MRSA exposure. Most of them utilized nasal drop-in only (Broens et al., 2012; Jouy et al., 2012; Szabó et al., 2012; Verstappen et al., 2014) or the combination with skin (Crombé et al., 2012) or gastrointestinal (Moodley et al., 2011) inoculation. The dosage used here was between 10^7^ cfu/mL (Verstappen et al., 2014) and 10^8^ cfu/mL (Moodley et al., 2011; Broens et al., 2012; Crombé et al., 2012; Szabó et al., 2012), whereas this dosage did not always result in a successful colonization (Moodley et al., 2011; Broens et al., 2012). Jouy et al. (2012) used a MRSA concentration of 10^4^ cfu/mL for the nasal drop-in without resulting in persistent nasal colonization. Oral inoculation of 50 mL containing 10^9^ cfu/mL resulted in a colonization but also in death of most of the pigs induced by pneumonia (Broens et al., 2012). In our model, as expected for a colonization in contrast to an infection, no clinical signs occurred. The usage of a high dosage in combination with one specific inoculation method to reach a stable colonization probably falsifies the transfer of results to field conditions. According to Crombé et al. (2012), the need of high dosages of MRSA could result in a greater transmission between animals due to a higher amount of MRSA inoculated animals compared to animals in the field. Although we used a lower airborne MRSA concentration compared to Szabó et al. (2012) using the nasal drop-in method with 5 × 10^8^ cfu/animal, our animals showed a higher colonization status at all time points. A model developed by Moodley et al. (2011) simulated a natural colonization by an experimental vaginal colonization of sows leading to stable MRSA colonized piglets over 4 weeks.

Furthermore, some studies used piglets with absent nasal microbiota (Verstappen et al., 2014) or by antibiotic treatment (Moodley et al., 2011) influenced natural nares microflora to enhance the ability for MRSA to colonize pigs. By contrast, our model used conventional raised, non-antibiotic treated animals. There is some evidence that a higher MRSA dosage for colonization is required in the presence of MSSA due to occupied attachment sides. Several authors make MSSA carriage responsible for MRSA colonization failure (Broens et al., 2012; Jouy et al., 2012). Co-colonization experiments with MRSA and MSSA conducted by Verstappen et al. resulted in a statistically higher MSSA than MRSA colonization. However, the piglets used in our study harbored MSSA naturally. Nevertheless, in comparison with other colonization models, our dosage is lower compared to the other models described despite the natural MSSA carriage of our animals.

### Evaluation of the Airborne Colonization Model

The airborne way of exposure seems to be less artificial than the nasal drop-in method used in the other studies and imitates field conditions in a reasonable manner. In conventional pig farms, MRSA occurs in dust and was found in air samples regularly (Friese et al., 2012). According to Ferguson et al. (2016), the size of airborne particles depends on the collection points: In the barn air, MRSA was bound on particles larger than 5 μm originating from feces. By contrast, MRSA found in the surrounding of pig barns was bound on particles originating from feces or epithelial cells with less than 5 μm. These reports indicate an early deposition of large particles and a prolonged stay and, therefore, wider spread of smaller particles in the air. The particle size measured in our aerosol chamber was around less than 5 μm and, therefore, imitated the entry of MRSA in the barn *via* the airborne route very well. The mean concentration found in barn air according to Friese et al. (2012) was about 10^2^ cfu/m^3^. Within our study, in contrast, the identified dosage for a persistent colonization is much higher with 10^6^ cfu/m^3^ over 24 h. This could be due to various reasons: the duration of exposure for 24 h in the aerosol chamber is not comparable to the exposure duration of animals in conventional pigsties. There, piglets are exposed often to MRSA for the completely fattening period of 6 months. An increase of the exposure time could be a next step to investigate the temporal influence for MRSA colonization. All animals exposed to the MRSA concentration of 10^4^ cfu/m^3^ were MRSA positive directly after the exposure with a decrease of MRSA colonization over time. It is very likely that the MRSA colonization success would be bigger when exposed longer or repeatedly – similar to field conditions where often a continual MRSA load in the air exists. It is also important to note that farm animals are challenged by other factors that could influence the MRSA colonization success. Possible factors are antibiotic treatment, immunosuppression induced by stress, bacterial endotoxins, and harmful (ammonia, hydrogen sulfide, and hydrocarbons) gases in barn air. Therefore, more research on these topics needs to be done. In addition, the aerosolized MRSA suspension itself could be a potential reason for a lower colonization capacity. Crombé et al. (2013) postulated before that the preparation process of MRSA suspensions could influence the physiological state of MRSA and, therefore, the ability to colonize animals. Aside from that, the aerosolization procedure could influence the bacteria negatively or, the time of particle distribution through the whole aerosol chamber. However, in our study, there was no difference of MRSA viability between different measure points within the chamber since the concentration of airborne MRSA measured in the different heights *via* impingement was similar.

### Airborne MRSA Colonization as the Initial Transmission Route in Our Animal Model

A weakness of our animal model is the inability to differentiate between an exclusive airborne colonization and a colonization additionally caused by contact to MRSA-contaminated chamber surfaces or animals’ skin due to bacterial deposition. However, there are several indications for the assumption that the main and initial colonization way of the piglets was dominated *via* the airborne route rather than by contact to contaminated surfaces. Almost all animals of groups MD and all of HD had MRSA-positive skin swabs on days 1 and 3 after exposure. If direct contact to contaminated surfaces such as skin would be the main transmission way, the animals of the MD group should be MRSA positive for a longer time – especially on skin. However, at day 6 after the exposure, only one-third of the skin swabs were MRSA positive, whereas the animals’ skin of the HD group remain positive until the end of the experiment. Obviously, the presence of MRSA on skin within the MD group was not sufficient to act as a source for MRSA re-colonization by direct contact, e.g., nose-skin contact. In addition, nose swab samples were negative within the MD group at day 8 after the exposure. Thus, the initial MRSA exposure dosage in the air was most likely responsible for the stable MRSA detection on the animals’ skin within the HD group. A colonization *via* contaminated walls or ground floor inside the aerosol chamber seem rather unlikely as the MRSA load on the wall surfaces after exposure was very low. Whereas quantification of MRSA in both swabs taken of aerosol chamber walls was not possible for MD group, the sampled walls of HD group were quantifiable but in a low MRSA concentrations of 2 and 0.7 cfu/cm^2^. This surface concentration seems to be too low to act as a dominant source for MRSA transmission by direct contact. A further indication for an initial colonization *via* the airborne route is that our airborne MRSA concentration was lower compared to the necessary MRSA concentration for a successful colonization of pigs used in the nasal drop-in model. Therefore, it is very unlikely that deposited airborne MRSA on the animals’ skin was a sufficient source for MRSA colonization.

### MRSA Colonization and Contamination of the Animals

Another interesting point to discuss is the differentiation between a true colonization and a transient contamination of the animals.

We strongly assume that in the LD group, no colonization occurred: One piglet of this group showed one MRSA-positive nasal swab directly after the exposure. MRSA detection in the anterior nose at one time point is no evidence for a true colonization. The following sampling, this piglet became MRSA negative and remained negative. This underlines the finding (Angen et al., 2017) where nasal swabs were taken from persons after staying 1 h in a pig barn. Almost all persons were sampled MRSA positive; 48 h later, most of the persons were MRSA negative again. Becoming MRSA negative after a short time indicates a transient contamination, not a colonization.

We assume that the animals of the HD group were stable colonized. All skin and pharyngeal swabs were MRSA positive the whole time. MRSA was detectable over the observation period in 97.5% of the nasal, 96.3% of the conjunctival, and 92.5% of the rectal swabs. The quantification was possible for all kinds of swab samples the first days after exposure and decreased over time. Our data suggest a stable colonization of piglets occurred due to a high number of MRSA-positive swab samples per animal at the different sampling points until the day of necropsy.

For the MD group, we assume that the animals were transiently colonized. Eight out of nine animals (89%) were MRSA positive on the skin directly after the exposure. The following decrease of MRSA-positive skin swabs over the time suggests a contamination rather than a true colonization of the animals’ skin. For the skin, nasal as well as conjunctival swabs, it is difficult to distinguish between a true colonization and a transient contamination of the animals immediately after the aerosol exposure due to a possible direct deposition of aerosolized MRSA from air on these sampling sites. The steep decline of MRSA-positive conjunctival swabs from day 1 to day 3 after exposure as well as the sporadic detection of MRSA during the observation period suggests a contamination of the piglets’ conjunctiva. Not only the detection rate, also the MRSA load of the skin decreased during the following sampling time points, since quantification was possible for the first two observation points after exposure only. This could be an indicator for the absence of proliferation and, therefore, for a true colonization (Jouy et al., 2012). On the other hand, there was the recurrence of MRSA-positive skin swabs within the MD group from day 13 until the end of the observation period. The recurrence of MRSA-positive swab samples at the last sampling points after their absence at the samplings before was also seen for the nasal and pharyngeal swabs. This might be caused by recontamination due to other persistently MRSA-positive animals of the study group associated with MRSA contamination of the animals’ surrounding.

Methicillin-resistant *Staphylococcus aureus*-positive rectal swabs are a result of swallowed bacteria and, therefore, assigned to true colonization. The decrease of MRSA-positive rectal swabs within the first week after exposure indicates a temporary colonization. Two positive swabs of the rectum reoccurred at day 21 simultaneously to positive nasal and skin swabs. However, a quantification was not possible for rectal swabs, which indicates a low MRSA load there. The presence of MRSA in the pharynx can be attributed to true colonization rather than contamination. The number of positive samples decreased over time, which underlines a transient colonization. We suspect that the presence of MRSA-positive pharyngeal swabs at the end of the observation period is a consequence of recolonization, especially in context of the recurrence of other positive swab samples. However, due to the intensive cleaning of our experimental pig barn once a day, the MRSA load in the environment was reduced and, therefore, also its capacity to act as a source for MRSA spreading. It seems more likely that MRSA-positive animals contaminated the environment, since the number of positive tested environmental swabs increased similar to the number of positive animals. The animals with the numbers 63 and 58 of the MD group were MRSA positive on six out of nine sampling points and could therefore act as permanent carriers. Animal number 61 showed MRSA-positive nasal, rectal, and skin swabs at day 21. This may indicate that one animal was colonized stable in the MD group.

Statistical analysis show that the kind of swab sample does not influence the animals’ MRSA status when considering the whole observation period for determining their status. This is because almost all swab samples were MRSA positive directly after the exposure and, thus, all animals had a positive status. However, the MRSA status is significantly influenced by the different swab sample types when considering the sampling time point. This result underlines the distinction between true colonization and transient contamination. Directly after the MRSA exposure, almost all swabs of every animal were MRSA positive, but the number of positive swabs decreased over time since the sampling sites were probably only contaminated. However, specific sampling sites remained MRSA positive for a longer time. Those were favored MRSA colonization sites like the head’s mucosa. The nasal mucosa was the most preferred location for a MRSA colonization. This is also shown by the significant twofold higher detection rate of MRSA in nasal swabs compared to pharyngeal swabs within the HD group.

The results of the second MD group show the strong reproducibility of the transient experimental MRSA colonization of the piglets by exposing these animals to an airborne MRSA concentration of 10^4^ cfu/m^3^. This shows that our airborne colonization model gives reproducible results and is, therefore, a valid colonization model for further investigations.

### Spread of MRSA Into Organs and Tissues

As Crombé et al. (2012) already pointed out, the inability to distinguish between true colonization and transient contamination of animals is a well-known problem given the fact, that there are no defined criteria for colonization. They assumed true colonization when *post-mortem* isolation of MRSA in the animals’ throats was possible. This matches the findings of our persistent colonized HD group, where MRSA was present in the tonsils of all animals in rather high concentrations. In addition, in the HD group, MRSA could be found during the completely observation period while the bacterial load of MRSA decreased. Tonsils are the first line of defense targeting bacteria after nasal or oral uptake. Szabó et al. (2012) used nasal drop-in with a dosage of 10^8^ cfu/mL and had similar results concerning the tonsils. However, they found MRSA also in other investigated organs. The probable reason for limited spread of MRSA in organs of our animals is the usage of a lower MRSA dosage and the different exposure route.

The experimental exposure of the piglets to MRSA *via* the airborne route within our study also imitate a possible entry of airborne MRSA in pig barns. Sources could be neighbored MRSA-positive barns within the same farm or maybe other farms nearby. With our model, we were able to expose piglets to defined MRSA concentrations in the air in order to investigate the effect of specific airborne bacteria dosages. We achieved a stable, reproducible colonization of conventional raised, non-pretreated piglets *via* a natural like way of airborne exposure with a MRSA dosage that is lower compared to the already existing MRSA colonization models. In conclusion, the animal model reported in this study, is a useful tool to investigate the colonization kinetic in dependence of various factors influencing the MRSA colonization in future.

## Ethics Statement

This study was approved in accordance with the Directive 2010/63/EU and with the German Animal Welfare Act by the State Office of Health and Social Affairs Berlin, Germany (Landesamt für Gesundheit und Soziales Berlin, LAGeSo) under the registration number G 0403/12. The study was carried according to the institutional guideline for animal welfare of the Freie Universität Berlin.

## Author Contributions

UR and AF performed the study design. UR and AF designed the animal experiments. KR performed the laboratory work. AF and KR performed the sampling. RM performed the statistical analysis. KR evaluated the final data and wrote the manuscript. All authors have read and approved the final draft of the article.

## Conflict of Interest Statement

The authors declare that the research was conducted in the absence of any commercial or financial relationships that could be construed as a potential conflict of interest.
